# Use of Surface-Enhanced Raman Scattering (SERS) Probes to Detect Fatty Acid Receptor Activity in a Microfluidic Device

**DOI:** 10.3390/s19071663

**Published:** 2019-04-08

**Authors:** Han Zhang, Wei Zhang, Lifu Xiao, Yan Liu, Timothy A. Gilbertson, Anhong Zhou

**Affiliations:** 1 Department of Biological Engineering, Utah State University, Logan, UT 84322-4105, USA; han.zhang1985@aggiemail.usu.edu (H.Z.); wei.zhang@aggiemail.usu.edu (W.Z.); lfxiao0517@gmail.com (L.X.); 2 Department of Internal Medicine, University Central Florida, Orlando, FL 32827-7408, USA; Yan.liu@ucf.edu (Y.L.); Timothy.Gilbertson@ucf.edu (T.A.G.)

**Keywords:** Raman, SERS, microfluidics, fatty acid, GPR120, 4-mercaptobenzoic acid

## Abstract

In this study, 4-mercaptobenzoic acid (MBA)-Au nanorods conjugated with a GPR120 antibody were developed as a highly sensitive surface-enhanced Raman spectroscopy (SERS) probe, and were applied to detect the interaction of fatty acids (FA) and their cognate receptor, GPR120, on the surface of human embryonic kidney cells (HEK293-GPRR120) cultured in a polydimethylsiloxane (PDMS) microfluidic device. Importantly, the two dominant characteristic SERS peaks of the Raman reporter molecule MBA, 1078 cm^−1^ and 1581 cm^−1^, do not overlap with the main Raman peaks from the PDMS substrate when the appropriate spectral scanning range is selected, which effectively avoided the interference from the PDMS background signals. The proposed microfluidic device consisted of two parts, that is, the concentration gradient generator (CGG) and the cell culture well array. The CGG part was fabricated to deliver five concentrations of FA simultaneously. A high aspect ratio well structure was designed to address the problem of HEK cells vulnerable to shear flow. The results showed a positive correlation between the SERS peak intensity and the FA concentrations. This work, for the first time, achieved the simultaneous monitoring of the Raman spectra of cells and the responses of the receptor in the cells upon the addition of fatty acid. The development of this method also provides a platform for the monitoring of cell membrane receptors on single-cell analysis using SERS in a PDMS-based microfluidic device.

## 1. Introduction

The epidemic of obesity in the Western world has been attributed to several factors, including, but not limited to, an increase in sedentary lifestyle, socioeconomic factors, and perhaps most importantly, an overconsumption of nutrients. Of these nutrients, the ingestion of a high fat diet has been implicated as playing a particularly important role leading to the development of dietary-induced obesity [1,2,3]. Recent data from a variety of laboratories have attempted to elucidate the mechanisms underlying the recognition of dietary fat by the body as a means to eventually try and pharmacologically target the control of the fat intake. Cognate receptors for dietary fat include a class of fatty acid-sensitive G protein-coupled receptors [4,5]. The best described of these is the long chain polyunsaturated fatty acid receptor, GPR120, which mediates the taste (and post-ingestive) recognition of fat [6]. Mice lacking GPR120 (GPR120 knockouts) show a much-reduced behavioral response to fat (fatty acids) in the form of a reduced preference for linoleic acid and oleic acid [7,8]. The identification of GPR120 in taste cells within the oral cavity, coupled with the ability of taste cells to respond to the ligands of this receptor, is consistent with these receptors mediating the taste of fat [3]. Development of imaging capabilities to characterize the response and specificity of these receptors, their distribution, and the characterization of the effect of ligand binding will facilitate our understanding of the function of this important class of receptors.

Raman spectroscopy has proven itself as a non-invasive and sensitive detection technique in past decades. The Raman spectrum provides a molecular fingerprint that could be used to characterize specific molecules from complex samples. The frequency difference between the incident and emitted photons is described as the Raman shift, which is unique for individual molecules and is represented as “cm^−1^” (inverse of photon wavelength). The peak intensity of the Raman shift is related to the concentration of the molecules of interest. However, only a small fraction of light (~1 in 10^6^ photons) is scattered at optical frequencies, usually lower than the frequency of the incident photons (termed the “Raman effect”). As a result, Raman scattering suffers from low signal intensity. This problem can be overcome by using surface-enhanced Raman spectroscopy (SERS) [9,10]. SERS is a surface-sensitive technique that enhances Raman scattering by molecules adsorbed on a rough metal surface (Au and Ag nano-particles) or by nanostructures (plasmonic–magnetic silica nanotubes). The enhancement factor can be as much as 10^6^ to 10^14^. Therefore, SERS has been established as a powerful tool that can provide insight into the chemical components and into biomaterials with high sensitivity. Many excellent reviews describing SERS and its principles are available [11,12,13].

Microfluidics is currently a highly active field, particularly in the context of lab-on-a-chip (LOC) systems. The use of microfluidics to conduct biomedical and clinical research has a variety of advantages. It offers automation, miniaturization, high-throughput, nanoliter scaled sample volumes, and a large surface area-to-volume ratio [14,15]. Combining Raman with microfluidics allows for the accurate monitoring, analysis, and detection of a wide range of samples in micro-environments. So far, microfluidic platforms integrated with SERS for biochemical analysis have been extensively reported [16,17,18,19,20,21,22,23,24]. One of the major challenges of combining microfluidics and Raman spectroscopy is the strong background from substrates. Materials such as polydimethylsiloxane (PDMS), glass, and Poly(methyl methacrylate) (PMMA) are widely used for the fabrication of microfluidics because of their favorable optical properties and ease of bonding. However, they are Raman active polymers, which have their own Raman scattering signal. Many methods were recently reported to minimize the background from these materials. Substrate materials with a low Raman background such as MgF_2_ [25], CaF_2_ [26], and quartz [27] were employed as substitutes. However, the cost of these materials is high and the prototyping and bonding presents another technical challenge. Integrated laser fibers within microfluidics are also reported as an alternative method to avoid the background [28]. Nevertheless, the scale of the application remains limited and the fabrication cost high.

Here, we developed a 3D microfluidic device for the detection of fatty acid-induced GPR120 receptors using SERS. The device is comprised of two connected functional parts, namely: the concentration gradient generator (CGG) and the micro-well cell culture array. Compared with conventional dish culture methods, monitoring the cell response to fatty acids in our microfluidic device is high-throughput and low-cost. The CGG could deliver five different concentrations of FA simultaneously to 25 wells, eliminating the adaptation problems associated with repeated, sequential stimulations on individual cells. In addition, the entire device was several cm in length and the volume of each microwell was 3.14 µL, and hence, the fabrication cost and reagent consumption were much less. The 4-mercaptobenzoic acid (MBA) SERS probe was introduced so as to avoid the problems associated with the PMDS substrate background. The SERS spectra were collected to analyze the HEK cell GPR120 receptor response to different concentrations of FA. This method, for the first time, realizes the capability of the simultaneous measurement of the representative native cell peaks and the time-dependent fatty acid interaction with its specific receptor in a microfluidic environment, and also provides a platform for biochemical measurement using SERS in a PDMS-based device.

## 2. Materials and Methods

### 2.1. Materials

The 4-mercaptobenzoic acid (MBA), N-(3-dimethylaminopropyl)-N′-ethylcarbodiimide hydrochloride (EDC), N-hydroxysuccinimide (NHS), and doxycycline hydrochloride (DOX) were purchased from Sigma-Aldrich (St Louis, MO, USA). Blasticidin S HCl, hygromycin B, phosphate-buffered saline (PBS), and 0.5% trypsin–EDTA were purchased from Life Technologies (Carlsbad, CA, USA). Polyethylene glycol (PEG) products, thiol PEG acid (HS–PEG–COOH, MW 5000), and methoxyl PEG-thiol (mPEG–SH, MW 5000), were purchased from Nanocs Inc. (New York, NY, USA). The monoclonal GPR120 antibody was purchased from Santa Cruz Biotechnology Inc. (sc-99105). The linoleic acid was obtained from Sigma Chemical Co. (St. Louis, MO, USA). Ultrapure water (18 MΩ cm^−1^) was used in this work.

### 2.2. Device Fabrication

**Figure 1:**
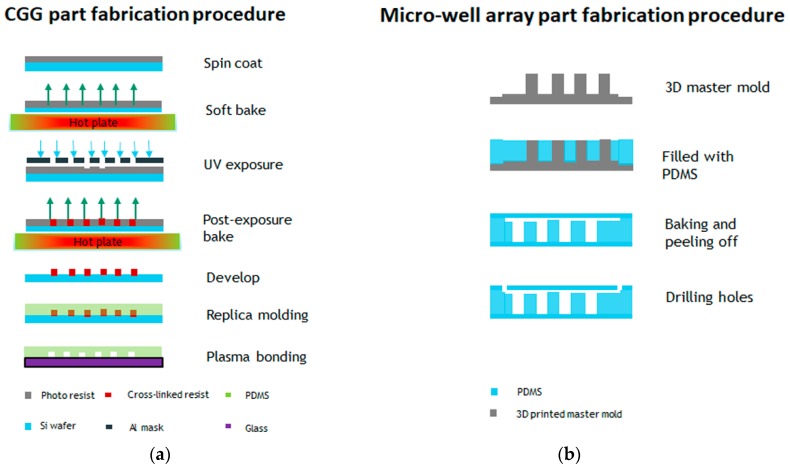

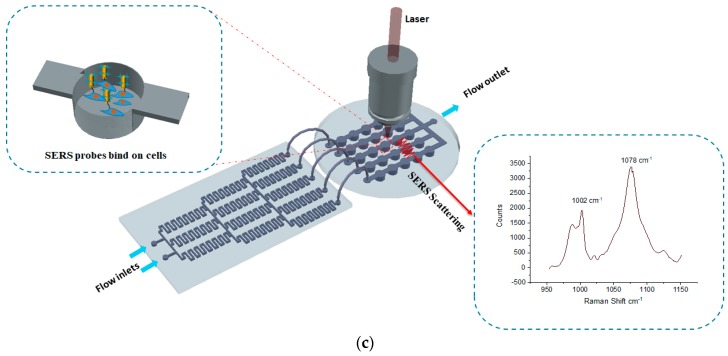
Fabrication steps of the microfluidic device and the experimental measurement setup. (**a**) Concentration gradient generator (CGG) part fabrication procedure. (**b**) Micro-well array part fabrication procedure. (**c**) Schematic of the experimental setup detection GPR120 receptors using surface-enhanced Raman spectroscopy (SERS).

The fabrication steps for both parts are illustrated in Figure 1a,b. The concentration gradient generator was fabricated using photolithography. The channel height and width were designed to 50 µm and 200 µm, respectively. The SU-8 2025 negative photoresist (Microchem^®^, Westborough, MA, USA) was spin coated on a three-inch diameter silicon wafer at a speed of 1800 rpm. The soft bake time was 3 min at 65 °C and 10 min at 95 °C. A transparency mask was printed at CAD/Art Services, Inc. (Portland, OR, USA) for the UV exposure. The post exposure bake time was 2 min at 65 °C and 7 min at 95 °C. PDMS soft lithography was applied in order to obtain the final device. The PDMS was mixed in a 10:1 ratio, stirred vigorously for 5 min, and then degassed for 30 min under dynamic vacuum to remove all air bubbles, and was cured at 60 °C for 2 h. The micro-well array part was replicated from a 3D printed master mold. The 3D master mold was fabricated using high-resolution acrylonitrile butadiene styrene (ABS), and a stereolithography (SLA) technique from 3D SYSTEM Inc. (Goleta, CA, USA). The total channel height was 1200µm (top flow channel 200 µm and bottom well 1000 µm) and the width was 1000 µm. Finally, the replicated PDMS layers were sealed by oxygen plasma etching at 50 W, for 30 s. The experimental setup for the detection of GPR120 by SERS is shown in Figure 1c.

### 2.3. Device Sterilization and Cell Seeding

The cell culture well array was assembled and sterilized by autoclaving for 20 min under 121 °C, after sterilization, ethanol was flowed through the channels at 30 μL/min for 30 min, followed by a PBS wash. The channels were tested for leakage by flowing food dye through the microchannel. Finally, the device was coated with fetal bovine serum overnight at 4 °C in fridge. The cells were trypsinized and fully suspended by vortex (Corning LSE) before injection to the microchannels. A 1 mL syringe (B-D^®^) was used for cell injection into the microfluidic well array culture chamber. The seeding density for all of the cell types was 5000 cells/cm^2^

### 2.4. Cell Preparation

Human embryonic kidney cells (HEK293) were kindly provided by International Flavors and Fragrances Inc. (IFF). The HEK293 cell lines transfected with an inducible GPR120 gene (HEK293-GPR120) were used in this context. The cells were grown in DMEM-GlutaMAX media (Life Technologies, 10569-010) supplemented with 10% Tet-free fetal bovine serum (Fisher, NC0290780). The cells were cultured in a humidified atmosphere at 37 °C with 5% CO_2_, and were passaged at over 75% confluence. Blasticidin S HCl (10 μg mL^−1^) and hygromycin B (100 μg mL^−1^) were added to the cell culture medium specifically for the maintenance of the inducible GPR120 gene.

### 2.5. In-Device Cell Culture and Fatty Acid Treatment

To evaluate the biocompatibility of the microfluidic device, HEK293-GPR120 cells were cultured in the PDMS device for a period of two days. The cells were injected manually to a microwell array culture chamber, and the entire device was placed in an incubator after seeding in a 5% CO_2_, 37 °C environment. To induce the expression of GPR120, a culture media with DOX at 0.5 μg mL^−1^ was used in the device for 48 h. The medium was refreshed every 24 h. The cell images were collected after the medium was replaced. After the 48-h incubation in the device, the CGG part of the device was connected to the well array part by tubing, and the linoleic acid and PBS buffer (1x) solution were injected into the CGG part through two inlets, using syringe pump at a speed of 50 μL /min for 5 min. After another 5 min treatment, the antibody-conjugated gold nanorods (AuNRs; SERS probe) in the culture medium were injected to wash out the previous solution, and were kept in an incubator overnight to ensure the completion of the GPR120 antibody and receptor binding. 

### 2.6. Synthesis of SERS Probe

The steps for the MBA-AuNR-antibody preparation are illustrated in Figure S1. First, 1 mL of a gold nanorod (AuNR) (Nanopartz Inc., Loveland, CO, USA) solution was mixed with an MBA solution (1 mM, 10 μL) and was incubated for 30 min. Solutions of HS–PEG–COOH (1 mg mL^−1^, 10 μL) and mPEG–SH (1 mg mL^−1^, 40 μL) were sequentially added to the nanorod solution and were incubated for 2 h. Then, the resultant solution was centrifuged (12,000 g, 15 min) to remove the excess PEG and MBA. The particles were re-suspended in water. Freshly prepared EDC (10 mM, 10 μL) and NHS (25 mM, 10 μL) solutions were mixed with the nanorod solution and were allowed to react for 30 min. The resulting solution was centrifuged, and the particles were re-suspended in PBS. At last, the anti-GPR120 antibody (0.2 mg mL^−1^, 10 μL) was added to the nanorod solution and was incubated for 1 h. Excess antibody was removed by centrifugation. The nanorods were re-suspended in PBS. The functionalized nanoprobe (MBA-AuNR-antibody) was stable in a solution for several days at 4 °C.

### 2.7. Raman Spectroscopy Measurement

The Raman spectra were measured using a Renishaw inVia Raman spectrometer, equipped with a 785 nm near-IR laser, which was focused through a 50× long working distance lens (Leica Microsystems). The laser intensity before and after travelling through the 50× objective was 110 mW and 29.4 mW, respectively (measured with LaserCheck, Coherent Inc., Portland, OR, USA). The grating used was 1200 L/mm. A silicon wafer was used for calibration before data collection (adjusted to 520.5 ± 0.1 cm^−1^ for the silicon peak). The HEK293 cells were cultured in the PDMS microfluidic device for 48 h, and the Raman measurement was conducted for the cells in a PBS buffer. The Raman spectra were collected over the range of 950 cm^−1^ to 1150 cm^−1^. The exposure time was 10 s for one accumulation at 100% laser power for all of the cell samples. The system spectral resolution was 1 cm^−1^. The cosmic rays in the raw spectra were removed using the “zap” function in Renishaw WIRE 3.4 software.

## 3. Results and Discussion

### 3.1. High Aspect Ratio Well Structure Evaluation

**Figure 2:**
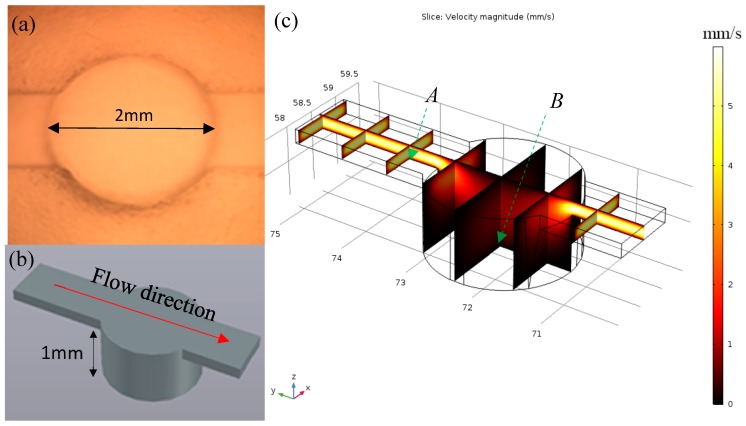
(**a**) Fabricated single well working unit; (**b**) AutoCAD 3D model of well unit; (**c**) COMSOL simulation of the flow rate distribution of the well unit (data were collected at cross-section plane A and B, 10 µm above boundary).

Because of the poor adhesion ability of HEK293 cell line, the cells were easily detached under shear flow in the microfluidic environment. To address this issue, the high aspect ratio micro-well structure was 3D printed and replicated by PDMS molding. The cell proliferation in the deep wells could prevent the detachment of cells during media refreshment and fatty acid injection. The dimensions of the well unit are illustrated in Figure 2a,b. The flow rate profile within the channel and microwell was simulated with COMSOL Multiphysics 5.2a (COMSOL Inc., Palo Alto, CA, USA). Three assumptions were made to mimic the actual flow situation, including the following: (1) the fluid is Newtonian, (2) no-slip boundary condition, and (3) the flow within the channel is incompressible. The 3D model was created initially in AutoCAD 2016 (Autodesk Inc., San Rafael, CA, USA), and then imported into the COMSOL library. The simulation was performed using physical interfaces laminar flow (spf) under the stationary study model. The inlet velocity flow was set to 40 µL/min. The simulation results are illustrated in Figure 2c. The flow speed reduced by 30.16 times from 1.93 mm/s to 0.064 mm/s (selected data collection points were 10 µm above the PDMS surface at cross-section plane A and B).

### 3.2. Evaluation of CGG

**Figure 3:**
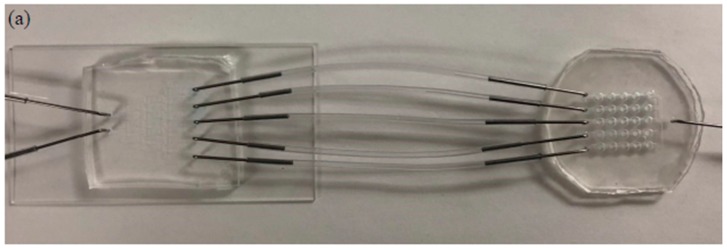

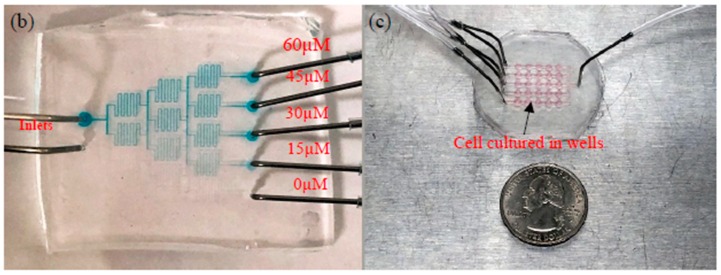
Current microfluidic device setup for GPR120 receptor detection; (**a**) two parts connected by tubing; (**b**) concentration gradient generator at a flow rate of 50 µL/min; (**c**) human embryonic kidney cell lines transfected with an inducible GPR120 gene (HEK293-GPR120) cell cultured in a well array.

The two fabricated parts and combined devices are illustrated in Figure 3. The blue color food dye was injected into the CGG part using a syringe pump at speeds of 200 µL/min, 100 µL/min, and 50 µL/min for 5 min. The gradient output streams were collected and evaluated by spectrophotometry. The results are summarized in Figure S2 and Table S1. The color intensities of the output streams were close to the corresponding standard solutions. The flow rate at 50 µL/min showed the most accurate concentration distribution. The errors could be caused by many factors, such as the uneven thickness of the photoresist on the fabricated master mold; trapped bubbles in the syringe, needle, or device during pumping; imperfection of hole punching for needle connection; and deviation of the tubing length. Based on the spectrophotometric results, the flow rate of 50 µL/min was chosen to generate the fatty acid concentration gradient for treatment.

### 3.3. Cell Culture in Device

**Figure 4:**
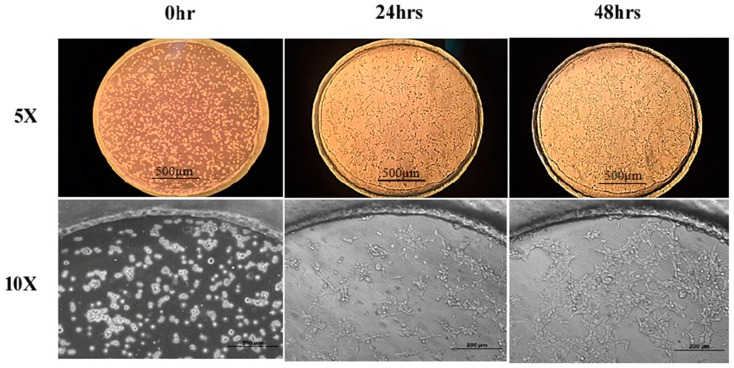
HEK293-GPR120 cells cultured in a well array for 48 h. Scale bars: 500 µm for 5× pictures, 200 µm for 10× pictures.

To evaluate the device biocompatibility, HEK293 cells were placed in a culture and were induced by DOX in the device for two days before the LA treatment. Figure 4 presents the results of the cells cultured in the microfluidic device at the time points of seeding, 24 h and 48 h under 5× and 10× magnification. It can be seen that the HEK293-GPR120 cells were grown and reached approximately 70% confluence on day 3. In addition, almost all of the cells attached to the substrate (Figure 4; 5× and 10×; 48 h), and relatively few suspended cells were observed. The results indicated that our device addressed the cell detachment issue and was biocompatible for the HEK293 cells.

### 3.4. SERS Measurement

Our previous research [29] is consistent with the interpretation that the FA treatment will increase the number of functional GRP120 receptors expressed on the cell surface, and hence would be expected to increase the MBA SERS signal upon binding between the anti-GPR120 antibody conjugated on nanorods and its antigen GPR120 on the cell surface. While the mechanism for this increased signal has not been elucidated, the binding of FAs to the receptor may induce a conformational change that facilitates the subsequent binding of the SERS probe or FAs themselves, and may facilitate the functional expression of previously quiescent receptors in the membrane. SERS has been proven to have the ability to detect the conformation change of DNA and protein receptors by several groups [30,31,32,33].

**Figure 5:**
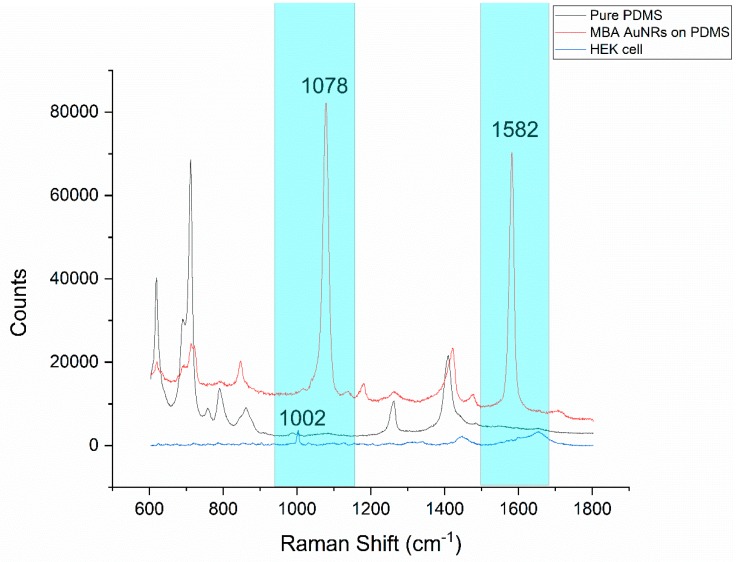
Raman spectra comparison, native HEK293 cell and polydimethylsiloxane (PDMS) with/without the presence of the 4-mercaptobenzoic acid (MBA)-gold nanorods (AuNR-antibody SERS probe. The spectral ranges with the MBA peaks and without the PDMS peaks are highlighted with cyan.

The main challenge of combining PDMS microfluidics and Raman spectroscopy is the strong background from PDMS (black curve, in Figure 5). Two dominant MBA specific peaks are presented at 1078 cm^−1^ and 1582 cm^−1^ (red curve, in Figure 5), which do not overlap with the PDMS background signal (cyan color highlighted zone in Figure 5). This is the major reason that the MBA was chosen as a Raman reporter molecule for the SERS probe construction in this work. Moreover, based on Figure 5, the counts of the PMDS baseline ranged from 2000 to 13,000, even in the area without visible peaks. HEK cell peaks with low counts would be hard to detect accurately in areas overlapping with the strong baseline. The HEK cell peaks with relatively high counts are marked in Figure S3. The peaks at 621 cm^−1^ and 1259 cm^−1^ are assigned to the C–C twisting mode of phenylalanine and amide III, respectively; both are protein bands. The peak at 787 cm^−1^ is assigned to DNA, it can be taken as a measure for the relative quantity of nucleic acids present. The peaks at 1301 cm^−1^ and 1447 cm^−1^ are assigned for the lipid and CH_2_ bending mode of proteins and lipids, respectively [34]. The peak at 1002 cm^−1^ represents phenylalanine. In most cases, the peak at 1002 cm^−1^ is one of the highest peaks, close to the MBA peak at 1078 cm^−1^ in a HEK293 cell spectrum [35,36,37,38]. In order to increase the signal-to-noise ratio, 1002 cm^−1^ was selected to represent a native cell peak as a comparison to the MBA peak (1078 cm^−1^). In addition, as shown in Figure S3, the other MBA SERS peak at 1582 cm^−1^ was much weaker and overlapped with the two strong peaks of amide II (1557 cm^−1^) and amide I (1656 cm^−1^) from the cells, which made it impossible to be accurately analyzed independently. Statistically, compared with the control group (normal culture), the average counts from the treatment group (treated with 60 µm linoleic acid, followed by incubation with the MBA-AuNRs-antibody) were increased from 780 (standard deviation (SD) = 204) to 2872 (SD = 2914) for 1078 cm^−1^, and 1202 (SD = 184) to 2183 (SD = 1356) for peak 1582 cm^−1^, respectively. Apparently, a larger peak increment was observed at 1078 cm^−1^. Taken together, in this study, the Raman spectra were collected from a range of 950 cm^−1^ to 1150 cm^−1^. This selected scanning range brings the opportunity to monitor the responses from the native cell peak (1002 cm^−1^) and the SERS peak (1078 cm^−1^) from the SERS probe conjugated with a specific antibody to receptor GRP120. By monitoring these peak changes (primarily intensity changes), the influence of fatty acids on the cell itself and the interaction between fatty acid and its receptor GPR120 could be detected simultaneously in the selected scan range.

**Figure 6:**
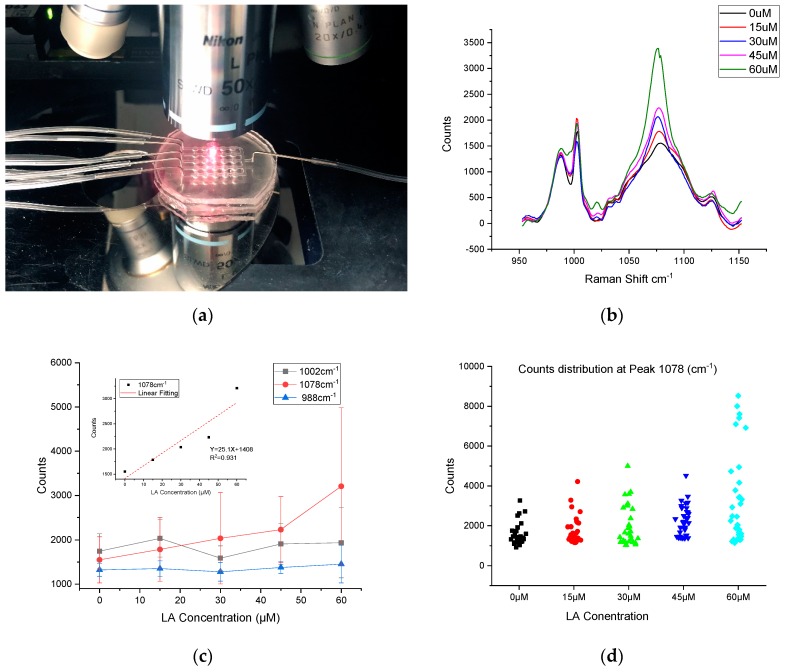
SERS measurement setup and results (**a**) SERS measurement of HEK293-GPR120 cells cultured in the microfluidic device. (**b**) Mean Raman spectra of HEK293-GPR120 cells treated with different concentrations of linoleic acid (*n* = 30–35). (**c**) Mean counts and standard deviation of HEK293 cell Raman spectra at peak 988 cm^−1^, 1002 cm^−1^, and 1078 cm^−1^ treated with different LA concentrations. Inset: relationship between SERS intensities at 1078 cm^−1^ peak and LA treatment concentration. (**d**) Counts distribution of the samples with different LA treatment concentrations.

The last step in this approach was to examine the cell response to the different concentrations of linoleic acid (LA) introduced to a cell culture device (Figure 6a). The HEK293-GPR120 cells were seeded and induced by DOX for 48 h. Different fatty acid concentrations were applied to different channels of microfluidic wells (0 µM, 15 µM, 30 µM, 45 µM, and 60 µM), the treatment time was 5 min and the unbound LA was washed off by injection of the media. After injection of the MBA-AuNRs-antibody SERS probes, the device was put back into the incubator for 12 h before the SERS measurement. The stability and reproducibility of the MBA SERS signal were evaluated before the SERS cell assay. The MBA-AuNRs were pipetted onto an MgF_2_ slice and left to dry for 30 min. The SERS peaks at 1078 cm^−1^ were collected from eight different spots (five replications at each spot) using a long working distance objective (50×). The result is shown in Figure S4; the counts of the parallel tests from the same spot were close to each other (Figure S4a). However, a large variation of counts was also observed at different collection spots (Figure S4b). The reason for the different peak heights in Figure S4b is because the SERS measurement is highly sensitive to the local environment that the incident laser interacts with (e.g., nanoparticle intensity, incident laser angles, and so on [11,12,13].

The SERS measurement of the cells in response to the different concentrations of LA is given in Figure 6b. Thirty-five spectra were collected from each treatment group; each spectrum was collected from different individual cell. Seven cells were randomly selected from each well (five wells for each concentration). The baseline was removed using built-in functions of the WIRE3.4 software. The arithmetic mean value of the counts from all of the samples was used to evaluate the correlation of the MBA peak to the LA treatment concentration. It was found that the SERS peak intensity (at 1078 cm^−1^) was positively correlated to the LA concentration, validating our previous observation that was conducted in a culture dish [29]; while the heights of the two peaks at 988 cm^−1^ and 1002 cm^−1^ appeared independent of the LA concentration (Figure 6c). The 1078 cm^−1^ average peak counts were 1549.39, 1784.20, 2035.73, 2232.12, and 3207.53 for LA treatment concentration 0 µM, 15 µM, 30 µM, 45 µM, and 60 µM, respectively. The SERS intensities at 1078 cm^−1^ vs. the LA concentration are plotted in Figure 6c. A linear (R^2^ = 0.931) increase in the 1078 cm^−1^ intensity was observed with the increased LA concentrations. These results also demonstrated the potential use of SERS to study the dynamic process of the biomolecules in single living cells. The relative standard deviations (RSD) of the sample spectra counts for different LA treatment groups—0 µM, 15 µM, 30 µM, 45 µM, and 60 µM—were 15.9% 22.0% 16.9% 23.7%, and 29.4% for peak 1002 cm^−1^, and 31.1% 40.9% 42.5% 33.3%, and 70.8% for peak 1078 cm^−1^. This large RSD at peak 1078 cm^−1^ could be caused by the dispersive counts distribution of the spectra collected from the cell surface (Figure 6d). Notably, in the 60 µM group (Figure 6d), two apparent populations are clearly visible. It is unclear why this happened. This phenomenon could be accounted for in a couple of ways, namely: the cell-to-cell variation of the in response to high concentrations of FA (e.g., 60 µM); or some cells expressing more GPR120 receptors would have a greater likelihood of being bound with anti-GPR120 SERS probes, leading to higher counts of signal. One-way Analysis of variance (ANOVA) was conducted with the confidence level of 95% (Figure S5), the Raman counts of 60 µM vs. 45 µM, 60 µM vs. 30 µM, 60 µM vs. 15 µM, 60 µM vs. 0 µM, and 45 µM vs. 0 µM were found to be significantly different. However, groups 45 µM vs. 30 µM, 45 µM vs. 15 µM, 30 µM vs. 15 µM, 30 µM vs. 0 µM, and 15 µM vs. 0 µM were not significantly different. The results of the non-significant differences can be caused by the close mean value of the paired groups and the relatively large RSD due to the non-uniform dispersive counts distribution. The ANOVA results also suggested that the cell responses were not strong enough to distinguish the corresponding concentration range (0–45 µM) of LA treatments with the increment of 15 µM, especially, under a large RSD. The counts distribution for each sample from the different treatment groups were demonstrated in Figure 6d. The sampling locations on the cell surface were randomly selected during the SERS data collection. Although there was a trend that higher LA concentrations lead to higher SERS responses, the variation of the SERS peak intensity for the same LA treatment concentration can be appreciable because of the cell’s non-uniform response to LA. For example, several high counts (7000–9000) were observed at the channel treated with 60 µM LA, on the other hand, several samples in each group had extremely low or even no detectable response to the treatment. The possible reasons could be that (1) some cells were resistant to the LA treatment, and a low or no SERS signal would be detected while focusing anywhere on these cell surface. (2) Not all of the GPR120 receptors on the HEK-GPR120 cells were activated by the LA treatment, thus the active zone may be only on certain sub-regions of the cell surface.

**Figure 7:**
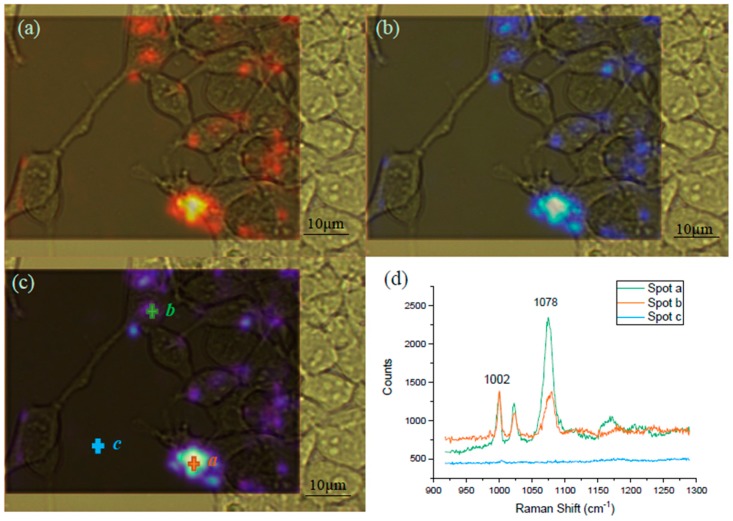
Raman mapping of the HEK293-GPR120 cells at the native cell peak 1002 cm^−1^ and the SERS peak 1078 cm^−1^. The framed areas were selected for the mapping of both the 1002 cm^−1^ (**a**) and 1078 cm^−1^ (**b**) peaks. Combination (**c**) of the spectral images at 1002 cm^−1^ (**a**) and 1078 cm^−1^ (**b**). The red (**a**) and blue (**b**) color intensities were positively correlated to the peak counts. Spectra from spot a, b, and c (**d**), Scale bar: 10 µm. The mapping spectrum scanning range was from 920 cm^−1^ to 1285 cm^−1^, 100% laser power, and 10 s exposure time. LA concentration: 60 µM.

Raman spectral mapping was employed for the visualization of the GPR120 receptor distribution on the single HEK293 cell surface (Figure 7). The mapping was conducted using HEK293-GRP120 cells cultured on MgF_2_, induced by DOX for 48 h, followed by the addition of 60 µM LA for 5 min, before being incubated with the SERS probe for 12 h. The blue color intensity was proportional to the SERS signal at 1078 cm^−1^ from the MBA reporter molecules. Three spots (a, b, and c) in Figure 7c were randomly selected to show the spectra at these locations. It was found that no SERS signal was detected at the area without cells attached (spot-c), the stronger color intensity (spot a) exhibits the higher color SERS peaks (spot b). These mapping results demonstrate that the MBA SERS probes were bound on the surface of most cells, however, the distribution of the bound receptors was not uniform over the entire cell surface. If a spectrum sample was collected from the cell area with no bound SERS probe, no detectable SERS signal would be seen. If the laser spot partially covered the activated area during the spectra collection, the signal can be extremely low. This diverse signal variation may explain the observation in Figure 6d. 

The spectra comparison between the signals (950 cm^−1^–1150 cm^−1^) acquired in the RDMS microfluidic wells and in the conventional dish culture on the MgF_2_ slices is illustrated in Figure S6. The PDMS substrate would cause a Raman laser power attenuation while laser travels through the top PDMS layer. The average counts of peak 1002 cm^−1^ and 1078 cm^−1^ declined to approximately 45.9% and 56.8%, respectively, compared to those obtained from the cells cultured on the MgF_2_ slice. However, the signal attenuation in the device was acceptable (due to two well defined MBA SERS peaks), and the intensity detected from the PDMS device was still adequate for analysis.

Our results are consistent with the interpretation that the fatty acids alone do not alter the surface structure of the cells, as noted from the lack of effect on the cell-related peaks. Instead, the fatty acid, as expected, appears to preferentially bind with the GPR120 receptor. It is clear that it was possible, using this approach, to monitor the interaction between the fatty acid and its cognate receptor by assessing the SERS peak from the anti-GPR120 antibody conjugated SERS probe. Prior to the addition of the SERS probe, the fatty acid binds a specific, though presently undetermined, region of the GPR120 receptor. Anti-GPR120 antibody (which is conjugated on the SERS nanorods surface) recognizes the extracellular epitope of the GRP120 receptor. The addition of the SERS probe and the subsequent antibody-antigen binding would lead to the conformation changes of the fatty acid and/or GPR120 receptor or their complex, which, in return, alters the local environment of the exposed SERS probe. The local environmental changes would give rise to the SERS peak changes. Simply, the more fatty acid molecules added, the more GPR120 receptors would be bound, thus the more SERS probes would recognize GPR120 receptors. Thus, this would be reflected in the higher SERS peaks. More experiments are needed in order to elucidate the specific mechanisms of interaction. Importantly, these series of experiments represent the first successful simultaneously monitoring of Raman spectra of cells (1002 cm^−1^) and the receptor-fatty acid interaction (1078 cm^−1^) in cells using a fabricated microfluidic device.

## 4. Conclusions

In this study, a PDMS integrating a concentration gradient generator (CGG) with the capability of delivering five concentrations of LA at a time and a micro-well array cell culture chamber was fabricated and tested in the detection of the presence of the GRP120 receptors and its interaction with fatty acid. The concentration generated in each CGG channel in the microfluidic device was evaluated by spectrophotometry with food dye. A 3D printed master mold was used for the fabrication of high aspect ratio micro-well cell culture array, to minimize the problem of HEK293 cell detachment under flow shear. The cell was cultured and induced in the device by DOX, for 48 h before the Raman measurement. An MBA-AuNR-GPR120-antibody-based SERS probe was constructed and employed to detect the GPR120 expression on the single HEK293 cell surface and the SERS probe, also to effectively avoid the interference from the PDMS background signal, when the appropriate spectral scanning range is selected. This microfluidic device, for the first time, achieves a simultaneous measurement of the representative Raman peaks from cells and the fatty acid interaction with specific receptor. Using this device and the SERS probes, we evaluated the dependence of the characteristic SERS peak height on the concentration of the fatty acid (LA). It was found that the SERS peak intensities were positively correlated to the LA treatment concentrations. The receptor distribution on a single cell level was also visualized by Raman mapping.

## Supplementary Materials

The following are available online at https://www.mdpi.com/1424-8220/19/7/1663/s1: Figure S1: Schematic of MBA-AuNR-antibody preparation steps; Figure S2: The concentrations gradient distribution using food dye with different injection flow rates; Figure S3: Representative Raman spectra of HEK293-GPR120 cell culture on a MgF_2_ slide (normal culture (blue) and cultured with MBA-AuNRs-antibody (treated with 60 µm linoleic acid) (red)); Figure S4: Stability and reproducibility of the MBA SERS; Figure S5: one-way ANOVA analysis of the counts at MBA peak 1078 cm^−1^ with different LA treatment concentrations, 95% confidence level (*n* = 30–35); Figure S6: Comparison of the peaks at 1002 cm^−1^ and 1078 cm^−1^ from HEK293 cells cultured in a dish and in a microfluidic device, the FA treatment concentration was 60 µM; Table S1: The relative percentage of the output streams with different flow rates (*n* = 3).
