# Supplementary material for: Use of Surface-Enhanced Raman Scattering (SERS) Probes to Detect Fatty Acid Receptor Activity in a Microfluidic Device
Source: Sensors (Basel). 2019 Apr 8;19(7):1663. doi: 10.3390/s19071663 (PMC6480160; doi:10.3390/s19071663)
Supplement: Supplementary file 1 [file sensors-19-01663-s001.pdf]

Supplemental Materials

Use of Surface-Enhanced Raman Scattering (SERS) Probes to Detect Fatty Acid Receptor Activity in a Microfluidic Device

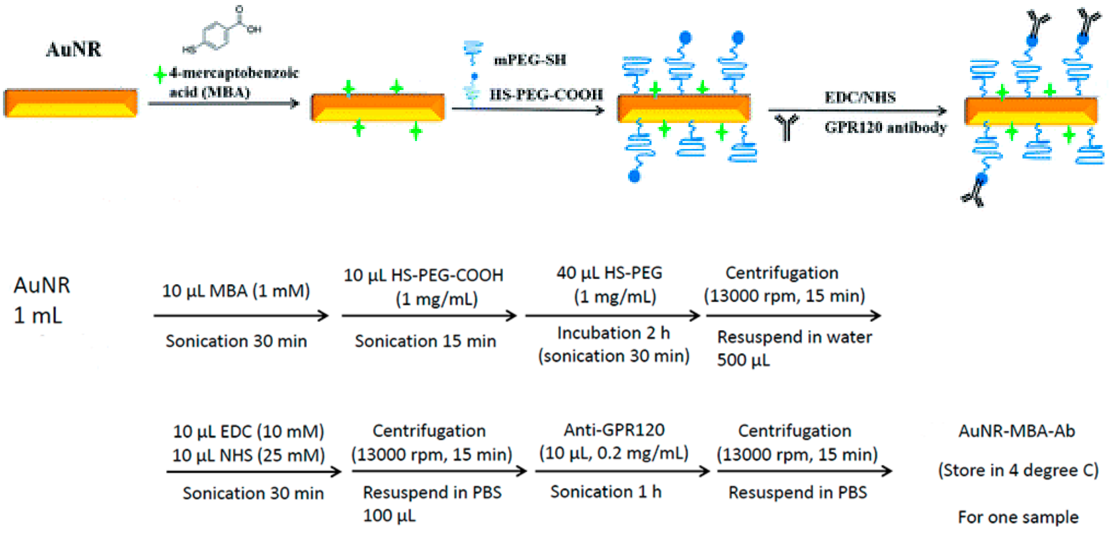

Figure S1. Schematic of MBA-AuNR-antibody preparation steps.

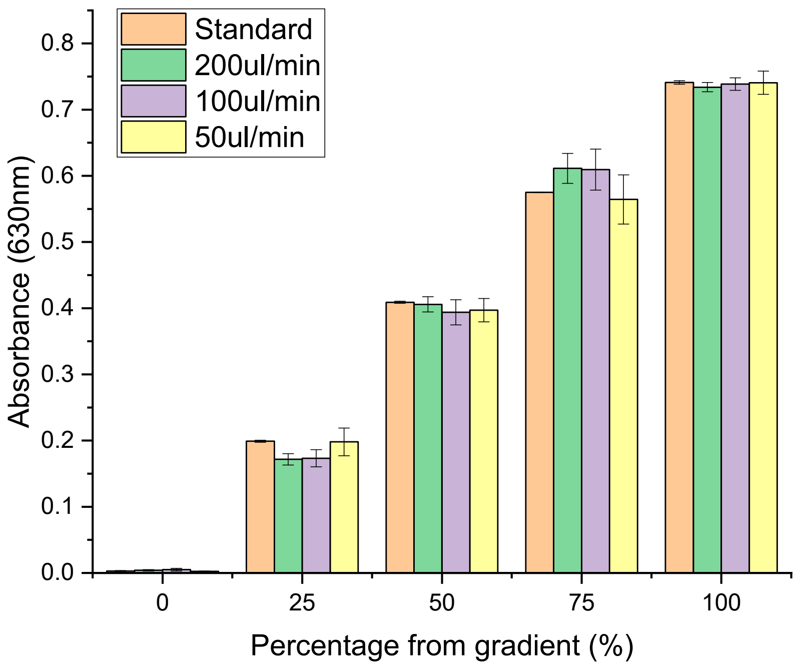

Figure S2. The concentration gradient distribution using food dye with different injection flow rates (200 µl/min, 100 µl/min and 50 µl/min). The "Standard" stand for food dye prepared to target concentration.

Table S1. The relative percentage of output streams with different flow rates (*n*=3).

Expected percentage	Standard	200µl/min	100µl/min	50 µl/min
0	−1.04 ± 1.23	−1.48 ± 1.89	−1.35 ± 1.76	−1.71 ± 1.95
25	24.56 ± 1.10	21.17 ± 0.86	21.85 ± 0.82	24.72 ± 0.80
50	52.56 ± 1.10	52.79 ± 0.47	51.17 ± 0.54	51.62 ± 0.38
75	75.76 ± 1.23	80.59 ± 1.02	80.32 ± 2.17	74.23 ± 2.98
100	98.56 ± 0.95	97.16 ± 1.08	97.79 ± 0.77	98.06 ± 0.35

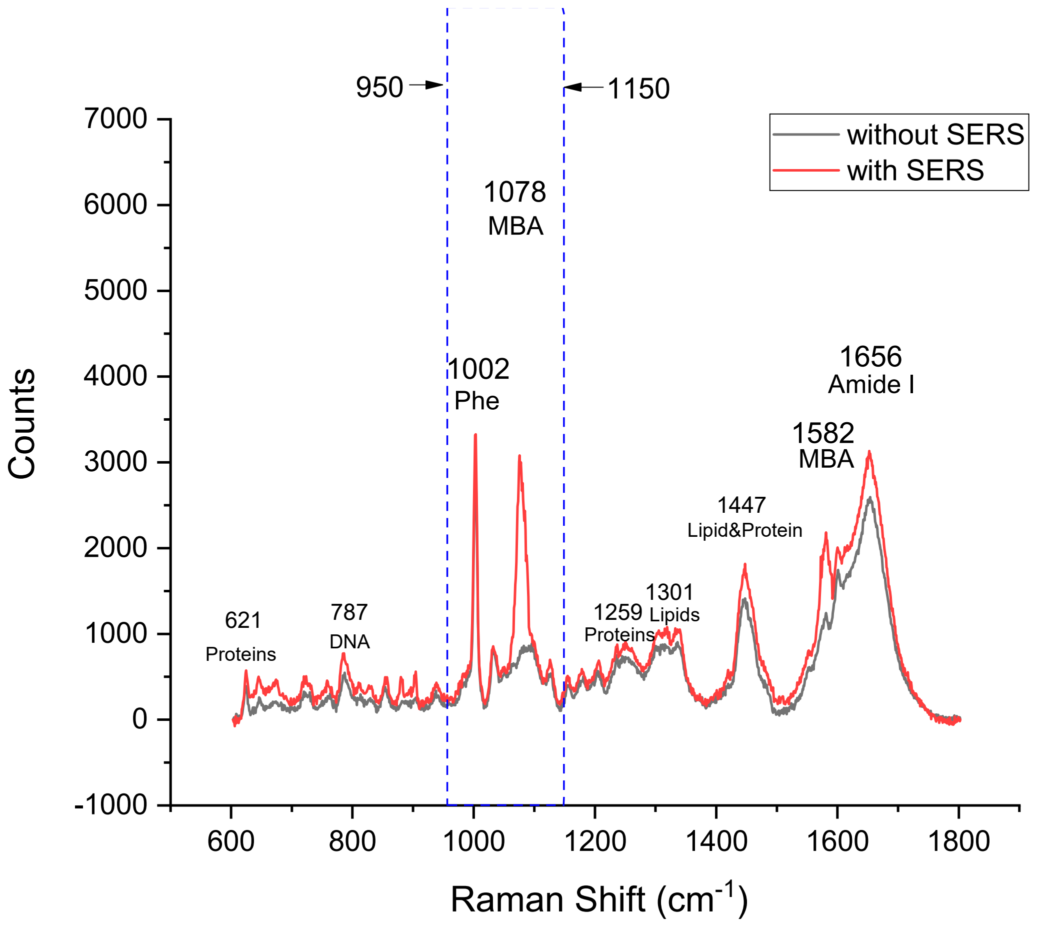

Figure S3. Representative Raman spectra of HEK293-GPR120 cell culture on MgF_2 slice. Normal culture (Blue) and cultured with MBA-AuNRs-Antibody (treated with 60μm linoleic acid) (Red). The spectra range 950 cm^{-1} ~ 1150 cm^{-1} was selected for our SERS measurement. Black and red shaded area are the standard deviations to the curve of corresponding colors (*n=10*).

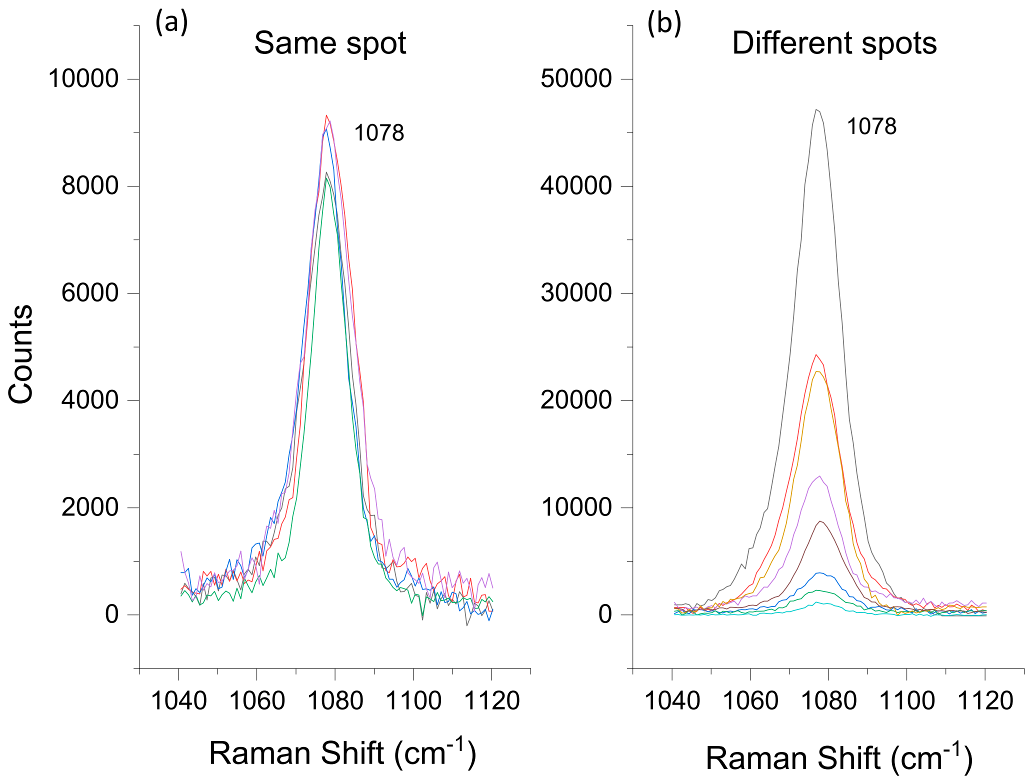

Figure S4. Stability and reproducibility of the MBA SERS, (a) MBA Spectra near 1078 cm^{-1} were collected for 5 times in a raw from same spot on MgF_2 substrate, (b) MBA Spectra near 1078 cm^{-1} were collected from different spots on the MgF_2 slice (Mean values of 5 replications).

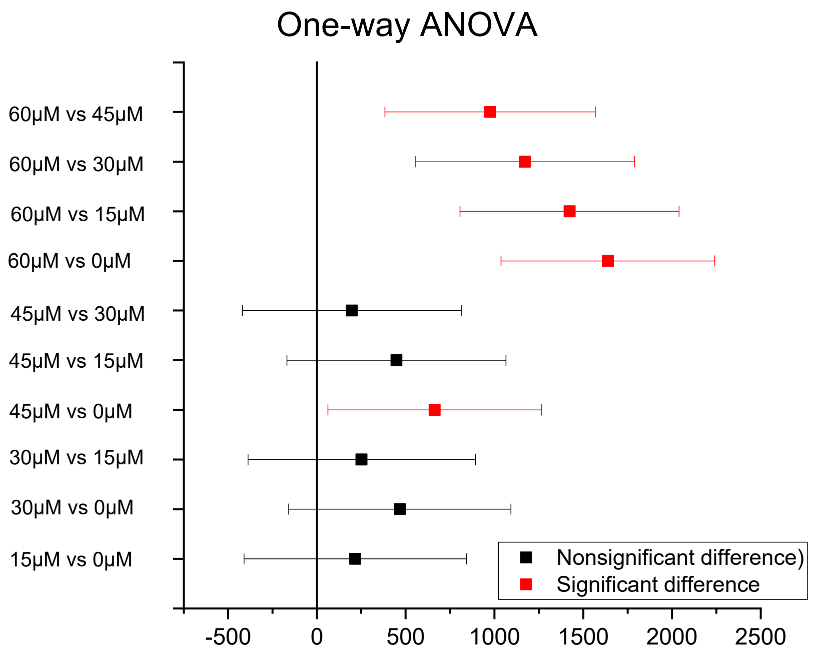

Figure S5. One-way ANOVA analysis of the counts at MBA peak 1078 cm^{-1} with different LA treatment concentrations, 95% confidence level (*n=30-35*).

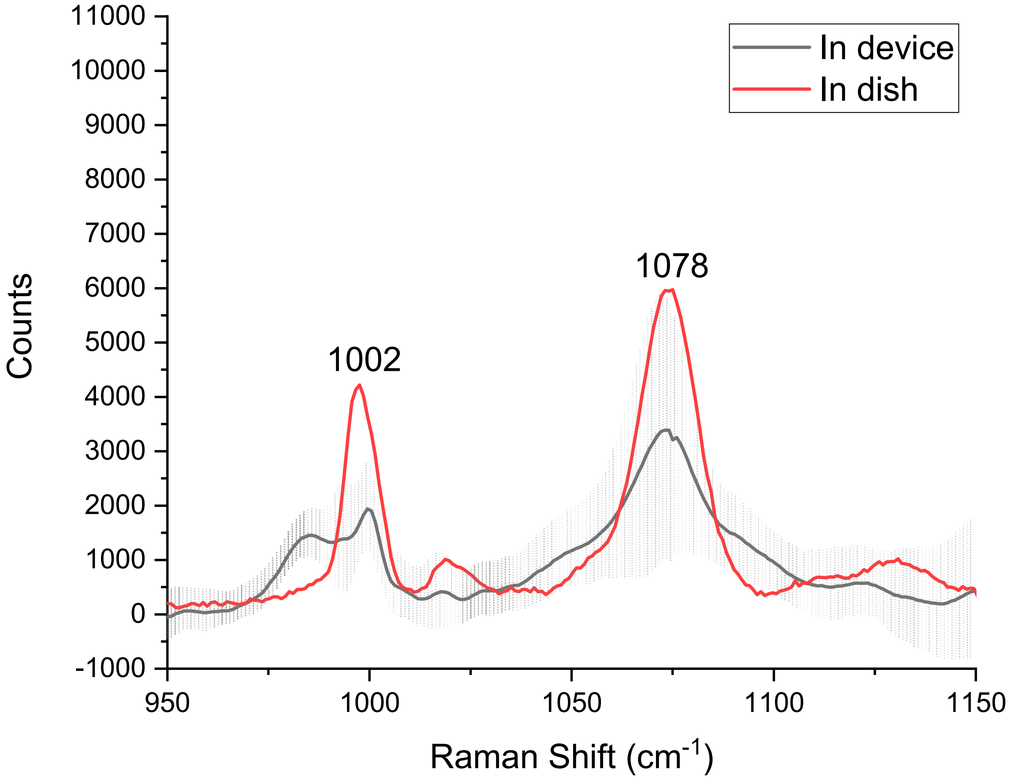

Figure S6. Comparison of the peaks at 1002 cm^{-1} and 1078 cm^{-1} from HEK 293 cells cultured in dish (red) and in microfluidic device (black) at the FA treatment concentration of 60 μM. The shaded areas are the standard deviations (*n=35* for device testing and n=25 for dish testing).
